# Determination of COVID-19 PCR positivity and mutation status in coronaVac vaccinated individuals in Turkey

**DOI:** 10.55730/1300-0144.5344

**Published:** 2022-01-01

**Authors:** Ahmet ÇALIŞKAN, Mücahit SEÇME, İlknur KALELI, Tuğba SARI, Sedef Zeliha ÖNER, Saniye KÜÇÜKAKIN YAKA, Büşra DÖNMEZ

**Affiliations:** 1 Department of Medical Microbiology, Faculty of Medicine, Pamukkale University, Denizli, Turkey; 2 Department of Medical Biology, Faculty of Medicine, Pamukkale University, Denizli, Turkey; 3 Department of Infectious Diseases and Clinical Microbiology, Faculty of Medicine, Pamukkale University, Denizli, Turkey

**Keywords:** SarsCov-2, CoronaVac vaccine, Sinovac, variant virus, Turkey

## Abstract

**Background/aim:**

CoronaVac is an inactivated virus-based COVID-19 vaccine used in Turkey and approved for emergency use by the World Health Organization (WHO). In this study, it was aimed to retrospectively evaluate the mutation status and clinical status in individuals who received two doses of CoronaVac vaccine and were infected with COVID-19 at least two weeks after the second dose.

**Materials and methods:**

164 people were included in the study and COVID-19 diagnosis and mutation analyses were determined by RT-PCR using the Bioseepdy SARS CoV-2 Double Gene RT-qPCR Kit and the Biospeedy SARS-CoV-2 Variant Plus kit in accordance with the protocol determined by the manufacturer.

**Results:**

116 (70.7%) UK (Alpha, B.1.1.7) mutation and 3 (1.8%) South Africa (Beta, B.1.351), Brazil (Gamma, P.1) mutations were determined in 164 double doses CoronaVac vaccinated patients; 45 (27.5%) patients were mutation negative. Nine patients (5.5%) developed pneumonia. Eight patients (4.9%) had CT findings compatible with corona virus infection. Seven (4.3%) of the patients received treatment in the intensive care unit, and 5 (3%) of the patients were intubated.

**Conclusions:**

In conclusion, people who receive two doses of CoronaVac vaccine can be reinfected with mutant viruses, vaccine significantly reduces the need for hospitalization, CT findings and intensive care.

## 1. Introduction

SARS-CoV-2 (severe acute respiratory syndrome coronavirus-2), which emerged in Wuhan, China in December 2019, is responsible for the COVID-19 epidemic that currently affects the whole world [1,2]. As of 30 June 2021, 181,344,227 cases have been detected all over the world, and 3,934,252 confirmed deaths due to COVID-19 have been reported globally^1^ . SARS-CoV-2 is a positive-sense, single-stranded RNA virus with a genome of approximately 30 kb in length and reported to contain 14 open reading frames encoding 27 proteins [3]. Since the beginning of the pandemic, SARS-Cov-2 genome sequencing studies have enabled genomic epidemiology studies of the origins, genetics and spread of viral variants of the COVID-19 disease. As of May 2021, more than 1.2 million SARS-CoV-2 genome sequences have been uploaded and shared through the GISAID (the Global Initiative on Sharing Avian Influenza Data) database^2^ [4]. These sequencing studies and the realization of new detections by monitoring variations in the SARS-CoV-2 genome ensured that genome changes have an observable impact on virus biology and a number of variants (VOCs, variant of concern) were quickly identified by late 2020 [4].

During the replication and spread of viruses, especially RNA viruses such as SARS-CoV-2, changes (mutations) develop in their genomes. The SARS-CoV-2 has a natural RNA repair mechanism and therefore accumulates mutations at a relatively slower rate than most other RNA viruses. Regarding the rate of change in the virus, it has been reported that it undergoes ~1.1 × 10−3 changes per site per year, corresponding to approximately one change every 11 days [5]. In all virus genomes sequenced to date, thousands of mutations have occurred since the beginning of the pandemic, resulting in thousands of new variants. Although many of the mutations do not have a full effect, some mutations can add some new characteristics to the virus, such as greater contagiousness and easy spread. These situations can trigger the selection of some variants and their becoming dominant after a certain period. However, more recently, several variants have been identified that appear to increase infectivity and potentially have an impact on disease severity^3^ . Several more rapidly spreading variants of the SARS-CoV-2 have recently been reported in the UK (Alpha, B.1.1.7), South Africa (Beta, B.1.351), Brazil (Gamma, P.1), and India (Delta, B.1.617), and determined in more 100 countries [6], including Turkey. B.1.1.7 originated in the UK, named as alpha variant, and was the first major SARS-CoV-2 strain of VOCs that was both more contagious and apparently more virulent [7]. The S protein (Spike) protein N501Y mutation increases the binding affinity of S protein and ACE2, while HV69–70del and Y144del deletions in the N-terminal domain (NTD) also play a role in ACE2 receptor binding or neutralizing antibody escape [7,8]. In recent studies, it has been reported new mutation situations that may result in the emergence of new and even more infectious sublineages of B.1.1.7. [7]. Variant viruses can trigger situations such as causing reinfection, evasion of the immune response, affecting different age groups, course of the disease, spreading and increasing mortality rates. In addition to all these, it is thought that it may reduce the effectiveness of the vaccine by avoiding the immune response that occurs after vaccination [9,10].

A large number of potential vaccines against COVID-19 have been developed at a rapid pace, and as of December 2020, extensive mass vaccination programs have been launched around the world [11]. As a result, it has been reported by the World Health Organization (WHO) that a total of 2,660,756,547 doses of vaccine have been administered as of 27 June 2021, since the vaccination started^1^. As of January 13, 2021, Turkey granted an emergency use permit to the inactivated virus-based ‘CoronaVac’ manufactured by the Chinese biopharmaceutical company Sinovac [11,12]. Furthermore, WHO has approved the CoronaVac vaccine for emergency use as of June 1, 2021^4^ . It was reported that CoronaVac clinical trial in Brazil showed lower efficacy numbers (50%) than in other countries such as Turkey (83.5%) and Indonesia (65.3%)^5^.

In Turkey, data for researchers about COVID-19 positivity and vaccines are limited. Thus, the available data from websites of the Ministry of Health were used. Daily cases, deaths, vaccinated people numbers were provided from the Ministry of Health. Healthcare workers are at a higher risk of exposure to coronavirus than other segments of society [13]. For this reason, health workers were the first group to be vaccinated in Turkey. In Turkey, COVID-19 vaccines started in mid-January, and vaccination studies of healthcare workers and the group over 65 years old were carried out quickly. Until the end of April, only CoronaVac vaccine was available in Turkey and the vaccination program was administered as two doses of 3mcg 28 days apart. It can be assumed that this situation provides us with maximum antibody formation and protection due to the vaccine in March-April and early May [14].

Data on the protection against variant viruses of CoronaVac is quite limited. This study aims to retrospectively evaluate the mutation status and clinical picture in individuals who are positive for COVID-19 after at least two weeks of the second dose of CoronaVac vaccine.

## 2. Materials and methods

### 2.1. Ethical considerations

Ethical permissions of this study were approved by both the Republic of Turkey Ministry of Health COVID-19 Scientific Research Evaluation Commission and the Pamukkale University Local Ethics Committee approval date: 8/06/2021; number E-60116787-020-60569, (No: 11).

### 2.2. Sample selection and viral RNA isolation and RT-qPCR

In this study, 164 cases who were diagnosed as COVID-19 positive at Pamukkale University Hospital between 21.03.2021 and 21.05.2021, although two doses of CoronaVac vaccine were completed, were included. Cases that completed at least 14 days or more after the second dose were included in the study. 14 days after the second dose of vaccine, these patients who were positive for COVID-19 were regularly reported to our laboratory by the Denizli Provincial Health Directorate for mutation study. Among the test results of the same patient, only one test result was included in the study. Vaccination data of Pamukkale University Hospital health workers were obtained from the Workplace Health and Safety Unit within our hospital.

The diagnosis of COVID-19 for the samples sent to our laboratory was determined by RT-PCR (Rotor Gene, Qiagen, USA) using Bioseepdy SARS CoV-2 Double Gene RT-qPCR Kit (Lot:2B01114NF25OG100-TK20, Turkey). This kit can be used on nucleic acid extracts obtained from nasopharyngeal aspirate/lavage, bronchoalveolar lavage, nasopharyngeal swab, oropharyngeal swab, and sputum samples with vNAT (viral nucleic acid buffer) extraction buffer. Rapid diagnosis with the kit is performed by one-step reverse transcription (RT) and real-time PCR (RT-qPCR) targeting the SARS-CoV-2 specific N and Orf1ab gene region. The kit targets the human RNaseP gene for sampling, nucleic acid extraction and inhibition control. The kit allows the reaction result to be reached in less than 50 min. Positive and negative patient results were determined in accordance with the protocol determined by the manufacturer. Subsequently, mutation analysis of the positive patients after vaccination was performed.

### 2.3. Mutation detection by RT-qPCR

In our study, mutation detection of positive cases was performed in RT-PCR (Rotor Gene, Qiagen, USA) device using Biospeedy SARS-CoV-2 Variant Plus kit (Cat No: BS-SY WCOR-402-250/BS-SY-WCOR-402-500/BS-SY-WCOR-402 1000, Turkey). The purpose of the kit is to identify variants arising from strains such as B.1.1.7 (UK), B.1.351 (South Africa), P.1 (Brazil), B.1.525 (UK and Denmark), B.1.526 (USA). “Bio-Speedy^®^ SARS-CoV-2 Variant Plus” kit, a one-step reverse transcription and real-time PCR (RT-qPCR) test, is designed for the qualitative detection of SARS-CoV-2 and variant strains in nasopharyngeal swab, oropharyngeal swab, nasal swab, nasopharyngeal aspirate, saliva and bronchoalveolar lavage specimens. Detection of mutations was performed on the basis of RT-PCR using the kit protocol specified by the manufacturer.

### 2.4. Statistical analysis

The data were analysed with the SPSS package program (IBM SPSS Statistics 25). Continuous variables are given as mean ± standard deviation and categorical variables as numbers and percentages.

## 3. Results

Between 28.03.2021 and 17.05.2021, 13,316 PCR test results were approved in Pamukkale University hospital. Of these, 1888 patient test results were detected as positive and 11,428 as negative. Among 1888 PCR-positive patients, 164 (8.7%) were double-dose vaccinated and 1724 (91.3%) unvaccinated. The vaccination rate among the COVID-19 PCR-positive patients was very low. Mutation analysis was performed in 434 of 1888 patients who were positive for COVID-19, and UK mutations were commonly detected. Of the studied patients, 296 (68.2%) were found as UK (Alpha, B.1.1.7), 6 (1.3%) South Africa (Beta, B.1.351), Brazil (Gamma, P.1), 132 (30.5%) mutation negative. Among 434 patients whose mutations were studied, 164 were patients who were positive at least 15 days after the double dose vaccination. Of 164 patients, 116 (70.7%) were found UK (Alpha, B.1.1.7) mutation, 45 (27.5%) were found mutation negative, 3 (1.8%) were found South Africa (Beta, B.1.351), Brazil (Gamma, P.1) mutations (Figure).

The mean age of 164 patients in the study was 57.7 years. Patients’ PCR tests were positive on average 51.8 days after the second dose of vaccine. Fifty-one patients (31.1%) were healthcare workers. Fifty-nine were males (36%) and 105 were females (64%). While no symptoms developed in 60 patients (36.6%), at least one symptom developed in 104 patients (63.4%). Nine patients (5.5%) developed pneumonia. Eight patients (4.9%) had CT findings compatible with coronavirus infection. Seven patients (4.3%) received treatment in the intensive care unit. Five patients (3%) were intubated. Four patients (2.4%) died. Of the 4 patients who died, 2 were UK mutations and 2 were nonmutant variants. These clinical data are given in Table.

## 4. Discussion

SARS-CoV-2 is a novel coronavirus that has infected more than 181 million people and caused approximately 4 million deaths as of the end of June 2021 worldwide^1^. Molecular genetic studies on the SARS-COV2 virus, such as genomic surveillance, differentiations, mutation detection, variant analysis, are of critical importance, especially in terms of diagnosis, treatment, and vaccination [15,16]. Further experimental research is needed to determine what changes the variants exhibit to evade immunity, the effect on sequence data and viral phenotypes. Studies are ongoing to assess whether VOCs reduce the efficacy and effectiveness of approved vaccines. Scientists use laboratory experiments and clinical data to assess whether individual mutations have an effect on vaccine efficacy^3^.

Safety, tolerability, and immunogenicity of CoronaVac vaccine in individuals aged 18–59 years have been reported with the data of randomized, double-blind, and placebo-controlled phase 1 and phase 2 trials [17]. As a result of the study, CoronaVac vaccine was shown to be well-tolerated and induced humoral response against SARS-COV-2, thus providing support for immediate use approval in Chine and phase 3 studies in clinical trials that are ongoing in Brazil (NCT04456595), Indonesia (NCT04508075), and Turkey (NCT04582344)^5^. According to results from phase 3 studies, CoronaVac vaccine efficacy against COVID-19 was found as 83.5% in Turkey^5^ [18] and 65.3% in Indonesia [19]. In Brazil, this efficacy rate was found to be 50.7%^5^. However, the efficacy rate of mRNA-based vaccines such as Pfizer/BioNTech and Moderna was found to be higher and reported as 95% [20] and 94.5% [21], respectively [22].

CoronaVac is a Vero cell-based, aluminium hydroxide-adjuvanted, β-propiolactone-inactivated vaccine dependent on the CZ02 strain of SARS-CoV-2 which was isolated from the bronchoalveolar lavage of a hospitalized patient and is firmly identified with the 2019-nCoV-BetaCoV Wuhan/WIV04/2019 strain^5^ [12]. Over time, mutations have triggered the original Wuhan virus to be replaced by a vastly different number of variants of concern viruses. Scientists are curiously working on how this will affect the effectiveness of existing vaccines. Nonetheless, various variations have raised worry because of transformations amassing especially in the S-quality and causing changes in the immunodominant epitopes of the trimeric spike protein. The D614G and B.1.1.7 variants of the SARS-COV-2 virus were neutralized by the vaccinees’ sera, demonstrating that these changes will probably not weaken the neutralizing antibody capacity induced by vaccination or natural infection. In any case, it ought to be noticed that the neutralizing titre of these sera was five-fold lower against the B.1.351 variation, which means that the amino acid changes accumulating in this variation are potentiating the escape of the infection from the humoral immunity. Nevertheless, more than 92% of vaccines demonstrated measurable neutralizing antibody titers against variant B.1.351. [23].

It is not currently known exactly how high neutralizing antibody titers are required against a particular virus variant for antibody-mediated protection against SARS-Cov-2 [23]. Although the exact efficacy of the CoronaVac vaccine against mutant viruses is not reported, it is thought that this vaccine reduces the course of hospitalization and severe illness. It is reported that the effectiveness of CoronaVac 14 days after the second dose in Chile was determined as 67% (65%–69%) effectiveness to prevent symptomatic COVID-19, 85% (83%–87%) effectiveness to prevent hospital admission, 89% (84%–92%) effectiveness to prevent intensive care unit (ICU) admission, and 80% (73%–86%) effectiveness to prevent death [24]. No study has yet been presented to evaluate the impact of the CoronaVac vaccine on the population level [14].

Although the publication of information about the protective effect of the Sinovac vaccine in Turkey is still limited, the first report on the effectiveness of the Sinovac vaccine on healthcare workers has been published recently [14]. Akpolat and Uzun (2021) have reported that the ratio of the death of healthcare workers to all residents decreased after vaccination. They have detected that vaccination indicates about 90% protection from death [14]. In our study, the number of patients who were positive for COVID-19 PCR in the specified date range was 1888. Mutation analyses of these 434 patients were completed and UK mutations were commonly detected. Mutation distributions were found to be 296 (68.2%) UK mutation, 6 (1.3%) South Africa-Brazil mutation, 132 (30.5%) mutation negative. Among those with positive PCR test, 164 were vaccinated with double dose CoronaVac vaccine and COVID-19 PCR tests were positive 15 days after the second dose. It was determined that 164 of 434 patients whose mutations were studied were positive 15 days after the double dose vaccine and these patients were included in the study. It was determined that 116 (70.7%) of the people who were infected after vaccinations had the UK mutation, 45 (27.5%) were mutation negative, and 3 (1.8%) had a South African-Brazil mutation. Among these 164 people, 51 were healthcare workers. Of these 164 people whose PCR tests were positive on average 51.8 days after the second dose of vaccine, 60 (36.6%) did not develop symptoms, while 104 (63.4%) developed at least one symptom. In addition, according to the data obtained, 9 (5.5%) of the patients developed pneumonia, while pneumonia did not develop in the vast majority (155 people, 94.5%). While 8 (4.9%) of the patients had CT findings compatible with coronavirus infection, no CT findings were observed in 156 (95.1%) patients. While 7 (4.3%) of the patients received treatment in the intensive care unit, 157 (95.7%) did not need intensive care. While 5 (3%) of the patients were intubated, 159 (97%) were not intubated. Of the 164 people included in the study, 4 died in our study group. The average age of those who are ex is quite advanced and determined as 82.2 ages in our study. Similar data were obtained in another study. At least one risk factor has been identified in all vaccinated deaths. Those who were vaccinated and died were determined to be older, and approximately 28% of those who died in the prevaccination period were younger than 55 years old, while the youngest of those who died after being vaccinated was 56 years old [14]. According to our results, while the rate of 1724 patients who were unvaccinated and whose COVID-19 test was positive among the applications made to Pamukkale University Hospital was determined as 12.9%, this rate was found to be 1.2% in double-dose vaccinated individuals. In this context, it was determined within the scope of this study that the Coronavac vaccine caused a serious decrease in the rate of positivity in the COVID-19 PCR test. All these results show us that, as a result of variant analyses in individuals who received two doses of CoronaVac vaccine and were infected with COVID-19, they can be reinfected with mutant viruses, but the vaccine significantly reduces the need for hospitalization, CT findings and intensive care. In another study in Turkey with CoronaVac, Akpolat and Uzun (2021) have reported that despite vaccination, healthcare workers continued to have COVID-19 infection, but less than the prevaccination period. They found that CoronaVac vaccine also reduced deaths by 95% [14].

## 5. Conclusion

In conclusion, the first data in Turkey regarding the mutation distributions in individuals who had received two doses of CoronaVac vaccine were shared within the scope of our study. In this study, it was observed that the need for hospitalization and intensive care for individuals who were vaccinated with CoronaVac vaccine decreased, but it was observed that reinfection with mutant viruses continued. Depending on the variety of variants that change day by day, planning detailed studies and determination of vaccine efficacy are important in terms of the up-to-dateness of vaccination studies. It is recommended to conduct more comprehensive studies with a larger population.

## Figures and Tables

**Figure:**
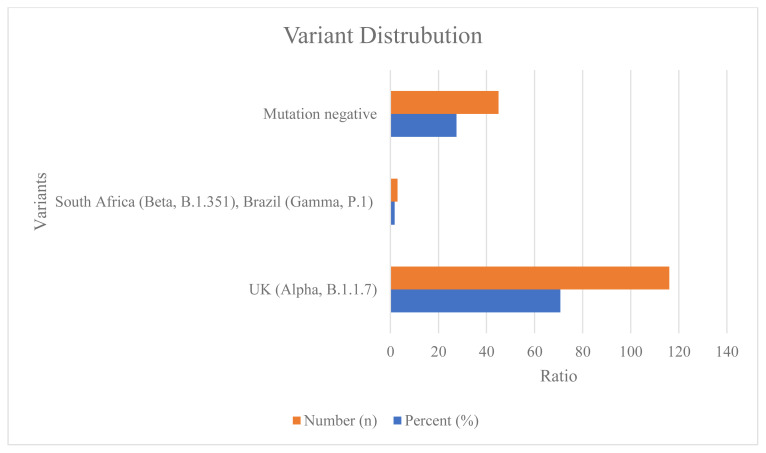
164 people were included in the study and COVID-19 diagnosis and mutation analyses were determined by RT-PCR. 116 (70.7%) were UK (Alpha, B.1.1.7) mutation, 45 (27.5%) were mutation negative, 3 (1.8%) were South Africa (Beta, B.1.351), Brazil (Gamma, P.1) mutations determined in 164 double doses CoronaVac vaccinated people.

**Table:** Clinical data status in 164 subjects who received two doses of CoronaVac vaccine and were reinfected with SARS-Cov-2 virus 15 days after the second dose CoronaVac vaccinated people.

	Symptom	Pneumonia	CT finding	Need for intensive care	Need for intubation	Ex
**Yes**	104 (63.4%)	9 (5.5%)	8 (4.9%)	7 (4.3%)	5 (3%)	4 (2.4%)
**No**	60 (36.6%)	155 (94.5%)	156 (95.1%)	157 (95.7%)	159 (97%)	159 (97%)

## Footnotes

1 WHO (2021) “WHO Coronavirus (Covid-19) Dashboard”, Available at: https://covid19.who.int/ . [Accessed 30 June 2021].

2 GISAID (2021). Global initiative on sharing all influenza data; Available from: https://www.gisaid.org/ . [Accessed 30 June 2021].

3 FIND Because Diagnosis Matters (2021). SARS-CoV-2 variants Reports. Available from: https://www.finddx.org/wp-content/uploads/2021/03/covid-variants-report-FINAL-12MAR2021.pdf [Accessed 11 March 2021].

4 WHO (2021). WHO validates Sinovac COVID-19 vaccine for emergency use and issues interim policy recommendations. Available at: https://www.who.int/news/item/01-06-2021-who-validates-sinovac-covid-19-vaccine-for-emergency-use-and-issues-interim-policy-recommendations [Accessed 30 June 2021].

5 WHO report. Background document on the inactivated vaccine Sinovac-CoronaVac against Covid-19. Available at: WHO REFERENCE NUMBER: WHO/2019-nCoV/vaccines/SAGE_recommendation/Sinovac-CoronaVac/background/2021.1. https://www.who.int/publications/i/item/WHO-2019-nCoV-vaccines-AGE_recommendation-Sinovac-CoronaVac-background-2021.1 [Accessed 1 June 2021].

## References

[1] Weston S Frieman MB COVID-19: Knowns, Unknowns, and Questions mSphere 2020 5 2 e00203 20 10.1128/mSphere.00203-20 32188753 PMC7082143

[2] Khan S Siddique R Shereen MA Ali A Liu J Emergence of a Novel Coronavirus, Severe Acute Respiratory Syndrome Coronavirus 2: Biology and Therapeutic Options Journal of clinical microbiology 2020 58 5 e00187 20 10.1128/JCM.00187-20 32161092 PMC7180238

[3] Wu A Peng Y Huang B Ding X Wang X Genome Composition and Divergence of the Novel Coronavirus (2019-nCoV) Originating in China Cell Host Microbe 2020 11 27 3 325 328 10.1016/j.chom.2020.02.001 32035028 PMC7154514

[4] Maxmen A One million coronavirus sequences: popular genome site hits mega milestone Nature 2021 593 7857 21 10.1038/d41586-021-01069-w 33893460

[5] Martin MA VanInsberghe D Koelle K Insights from SARS-CoV-2 sequences Science 2021 371 6528 466 467 10.1126/science.abf3995 33510015

[6] Rahman M Shirin T Rahman S Rahman MM Hossain ME The emergence of SARS-CoV-2 variants in Dhaka city, Bangladesh Transboundary and Emerging Diseases 2021 68 6 3000 3001 10.1111/tbed.14203 34170629 PMC8447378

[7] Shen L Bard JD Triche TJ Judkins AR Biegel JA Gai X Rapidly emerging SARS-CoV-2 B.1.1.7 sub-lineage in the United States of America with spike protein D178H and membrane protein V70L mutations Emerging Microbes & Infections 2021 10 1 1293 1299 10.1080/22221751.2021.1943540 34125658 PMC8238060

[8] Greaney AJ Loes AN Crawford KHD Starr TN Malone KD Comprehensive mapping of mutations in the SARSCoV-2 receptor-binding domain that affect recognition by polyclonal human plasma antibodies Cell Host Microbe 2021 29 3 463 476e6 10.1016/j.chom.2021.02.003 33592168 PMC7869748

[9] Jia Z Gong W Will Mutations in the Spike Protein of SARS-CoV-2 Lead to the Failure of COVID-19 Vaccines? Journal of Korean Medical Science 2021 10 36 18 e124 10.3346/jkms.2021.36.e124 33975397 PMC8111046

[10] Di Caro A Cunha F Petrosillo N Beeching NJ Ergonul O Severe acute respiratory syndrome coronavirus 2 escape mutants and protective immunity from natural infections or immunizations Clinical Microbiology and Infection 2021 27 6 823 826 10.1016/j.cmi.2021.03.011 33794345 PMC8007194

[11] Seyahi E Bakhdiyarli G Oztas M Kuskucu MA Tok Y Antibody response to inactivated COVID-19 vaccine (CoronaVac) in immune-mediated diseases: a controlled study among hospital workers and elderly Rheumatology International 2021 41 8 1429 1440 10.1007/s00296-021-04910-7 34109466 PMC8188953

[12] Gao Q Bao L Mao H Wang L Xu K Development of an inactivated vaccine candidate for SARS-CoV-2 Science 2020 3 369 6499 77 81 10.1126/science.abc1932 32376603 PMC7202686

[13] Adrielle Dos Santos L Filho PGG Silva AMF Santos JVG Santos DS Recurrent COVID-19 including evidence of reinfection and enhanced severity in thirty Brazilian healthcare workers Journal of Infection 2021 82 3 399 406 10.1016/j.jinf.2021.01.020 33589297 PMC7880834

[14] Akpolat T Uzun O Reduced mortality rate after coronavac vaccine among healthcare workers Journal of Infection 2021 83 2 e20 e21 10.1016/j.jinf.2021.06.005 34116073 PMC8187739

[15] Cyranoski D Alarming COVID variants show vital role of genomic surveillance Nature 2021 589 7842 337 338 10.1038/d41586-021-00065-4 33452508

[16] Shen L Bard JD Triche TJ Judkins AR Biegel JA Gai X Emerging variants of concern in SARS-CoV-2 membrane protein: a highly conserved target with potential pathological and therapeutic implications Emerging Microbes & Infections 2021 10 1 885 893 10.1080/22221751.2021.1922097 33896413 PMC8118436

[17] Zhang Y Zeng G Pan H Li C Hu Y Safety, tolerability, and immunogenicity of an inactivated SARS-CoV-2 vaccine in healthy adults aged 18–59 years: a randomised, double-blind, placebo-controlled, phase 1/2 clinical trial The Lancet Infectious Diseases 2021 21 2 181 192 10.1016/S1473-3099(20)30843-4 33217362 PMC7832443

[18] Tanriover MD Doğanay HL Akova M Güner HR Azap A Efficacy and safety of an inactivated whole-virion SARS-CoV-2 vaccine (CoronaVac): interim results of a double-blind, randomised, placebo-controlled, phase 3 trial in Turkey Lancet 2021 17 398 10296 213 222 10.1016/S0140-6736(21)01429-X 34246358 PMC8266301

[19] Fadlyana E Rusmil K Tarigan R Rahmadi AR Prodjosoewojo S A phase III, observer-blind, randomized, placebo-controlled study of the efficacy, safety, and immunogenicity of SARS-CoV-2 inactivated vaccine in healthy adults aged 18–59 years: An interim analysis in Indonesia Vaccine 2021 22 39 44 6520 6528 10.1016/j.vaccine.2021.09.052 PMC8461222 34620531

[20] Polack FP Thomas SJ Kitchin N Absalon J Gurtman A Safety and Efficacy of the BNT162b2 mRNA Covid-19 Vaccine New England Journal of Medicine 2020 31 383 27 2603 2615 10.1056/NEJMoa2034577 33301246 PMC7745181

[21] Widge AT Rouphael NG Jackson LA Anderson EJ Roberts PC Durability of Responses after SARS-CoV-2 mRNA-1273 Vaccination New England Journal Of Medicine 2021 7 384 1 80 82 10.1056/NEJMc2032195 33270381 PMC7727324

[22] Meo SA Bukhari IA Akram J Meo AS Klonoff DC COVID-19 vaccines: comparison of biological, pharmacological characteristics and adverse effects of Pfizer/BioNTech and Moderna Vaccines European Review for Medical and Pharmacological Sciences 2021 25 3 1663 1669 10.26355/eurrev_202102_24877 33629336

[23] Jalkanen P Kolehmainen P Häkkinen HK Huttunen M Tähtinen PA COVID-19 mRNA vaccine induced antibody responses against three SARS-CoV-2 variants Nature Communications 2021 28 12 1 3991 10.1038/s41467-021-24285-4 PMC8239026 34183681

[24] Jara A Undurraga EA González C Paredes F Fontecilla T Effectiveness of an Inactivated SARS-CoV-2 Vaccine in Chile New England Journal of Medicine 2021 2 385 10 875 884 10.1056/NEJMoa2107715d 34233097 PMC8279092

